# Trait divergence and opposite above- and below-ground strategies facilitate moso bamboo invasion into subtropical evergreen broadleaf forest

**DOI:** 10.3389/fpls.2024.1410372

**Published:** 2024-07-19

**Authors:** Hua Yu, Xingui Le, Josep Peñuelas, Jordi Sardans, Chaobin Xu, Yuxing Zou, Xue Zhang, Conghui Li, Zhenwei Mao, Dongliang Cheng, Quanlin Zhong

**Affiliations:** ^1^ College of Geographical Science, Fujian Normal University, Fuzhou, Fujian, China; ^2^ College of Geography and Oceanography, Minjiang University, Fuzhou, Fujian, China; ^3^ Department of Protection and Management, Administrative Bureau of Yangjifeng National Nature Reserve, Guixi, Jiangxi, China; ^4^ CSIC, Global Ecology Unit CREAF-CSIC-UAB, Bellaterra, Barcelona, Catalonia, Spain; ^5^ Ecological and Forestry Applications Research Center (CREAF), Campus Universitat Autònoma de Barcelona, Cerdanyola del Vallès, Barcelona, Catalonia, Spain; ^6^ Fujian Provincial Key Laboratory of Plant Ecophysiology, Fujian Normal University, Fuzhou, Fujian, China; ^7^ Key Laboratory of Humid Subtropical Eco-Geographical Process, Ministry of Education, Fuzhou, Fujian, China; ^8^ College of Tourism and Resources Environment, Zaozhuang University, Zaozhuang, Shandong, China

**Keywords:** moso bamboo (*Phyllostachys edulis*) invasion, trait divergence, phenotypic integration, conservation gradient, collaboration gradient, evergreen broadleaf forest

## Abstract

Understanding the invasion of moso bamboo (*Phyllostachys edulis*) into adjacent evergreen broadleaf forest based on functional traits is crucial due to its significant influence on ecosystem processes. However, existing research has primarily focused on above- or below-ground traits in isolation, lacking a comprehensive integration of both. In this study, we conducted a trait-based analysis including 23 leaf traits and 11 root traits in three forest types - bamboo forest, mixed bamboo and broadleaf forest, and evergreen broadleaf forest - to investigate trait differences, phenotypic integration, and above- and below-ground resource strategies in bamboo and broadleaf species. Our findings demonstrated significant differences in leaf and root key traits between bamboo and broadleaf species, strongly supporting the “phenotypic divergence hypothesis”. Bamboo exhibited stronger trait correlations compared to broadleaf species, indicating higher phenotypic integration. Above- and below-ground strategies were characterized by trade-offs rather than coordination, resulting in a multi-dimensional trait syndrome. Specifically, a unidimensional leaf economics spectrum revealed that bamboo with higher leaf N concentrations (LNC), P concentrations (LPC), and specific leaf area (SLA) adopted a “fast acquisitive” above-ground strategy, while broadleaf species with thicker leaves employed a “slow conservative” above-ground strategy. A two-dimensional root trait syndrome indicated a “conservation” gradient with bamboo adopting a “slow conservative” below-ground strategy associated with higher root tissue density (RTD), and broadleaf species exhibiting a “fast acquisitive” below-ground strategy linked to higher root N concentrations (RNC) and P concentrations (RPC), and a “collaboration” gradient probably ranging from broadleaf species with a “do-it-yourself” strategy characterized by high specific root length (SRL), to bamboo adopting an “outsourcing” strategy with thicker roots. In conclusion, key trait divergence from coexisting broadleaf species, higher phenotypic integration, and multi-dimensional opposite above- and below-ground resource strategies confer competitive advantages to moso bamboo, shedding light on the mechanistic understanding of its invasion into subtropical evergreen broadleaf forest and providing theoretical guidance for maintaining the stability of subtropical forest ecosystem.

## 1 Introduction

Plant invasion is a global concern due to its severe impact on threatening economic development, decreasing plant diversity, and impeding the regeneration of native vegetation (Luo et al., 2015; Shouman et al., 2020; Helsen et al., 2021; Juillard et al., 2024). Plant functional traits play a pivotal role in plants’ ability to acquire, utilize, and conserve resources, providing deeper insights into their responses to complex environmental changes (Violle et al., 2007; Helsen et al., 2021) and shedding light on the mechanisms of plant invasion (Osunkoya et al., 2014; Luo et al., 2015; Murphy et al., 2016; Mathakutha et al., 2019; Díaz de León Guerrero et al., 2020; Shouman et al., 2020; Castillo et al., 2021; Helsen et al., 2021; Palma et al., 2021; Rutherford and Archer, 2023). Specifically, investigations into trait values and phenotypic integration have documented their importance in conferring invasive species competitive advantages over native species (Osunkoya et al., 2014; Luo et al., 2015).

Regarding trait values, two opposing hypotheses have been put forward to explain successful invasion for invasive species. The “phenotypic convergence hypothesis” suggests that successful invaders possess traits similar to coexisting natives due to habitat filtering (Cornwell et al., 2006), facilitating invasive species preadaption to the local environment and thus invading native habitats more easily (Fridley and Sax, 2014), which has been supported by substantial evidence (Leishman et al., 2010; Lemoine et al., 2015; Lodge et al., 2018; Sodhi et al., 2019). Conversely, the “phenotypic divergence hypothesis”, based on limiting similarity, argues that successful invaders have distinct traits from coexisting natives, enabling them to occupy vacant niches (Ordonez, 2014). Traits such as higher leaf N and P concentrations (LNC and LPC), leaf area (LA), specific leaf area (SLA), leaf dry matter content (LDMC), mass-based net photosynthetic rate (A_mass_), specific root length (SRL), and root diameter (RD), as well as lower mass-based dark respiration rate (R_mass_) and leaf C:N ratio, have been found to benefit invasive species over native species (Luo et al., 2015; Murphy et al., 2016; Heberling and Mason, 2018; Mathakutha et al., 2019; Díaz de León Guerrero et al., 2020; Palma et al., 2021; Wang et al., 2021; Montesinos, 2022). Furthermore, phenotypic integration, the pattern of correlations among different functional, developmental, or genetic traits, is essential for alien plants to invade native communities successfully (Osunkoya et al., 2010, 2014).

However, there is still a lack of consensus and limited understanding of phenotypic integration. While some studies found stronger correlations of leaf traits in invasive vines in South-East Queensland of Australia and *Acer pseudoplatanus* in New Zealand compared to natives (Osunkoya et al., 2010, 2014; Shouman et al., 2020), others did not observe higher phenotypic integration in the invasive *Robinia pseudoacacia* than in the native *Sophora japonica* in Shandong province of China (Luo et al., 2015). Additionally, trait networks that represent correlations among multiple traits, and provide valuable information on overall trait correlation patterns, have been poorly explored in phenotypic integration (Michelaki et al., 2019). Moreover, existing studies have mainly focused on above-ground traits (e.g., leaf traits) and have paid less attention to below-ground traits (e.g., fine root traits) despite their crucial role in plant and ecosystem functions (Lambers et al., 2006). Therefore, it is imperative to integrate below-ground traits with above-ground traits in a unified framework to better understand plant invasion.

Furthermore, whether above- and below-ground traits coordinate in response to plant invasion remains unresolved. The plant economics spectrum (PES) posits that above- and below-ground traits are coordinated along a unidimensional axis, ranging from resource acquisition to conservation, owing to biophysical and evolutionary constraints (Reich, 2014). In other words, a unidimensional root economic spectrum (RES) was observed in conformity with leaf economic spectrum (LES), as below-ground roots are equivalent to above-ground leaves (Shen et al., 2019). However, conflicting findings suggest that plants may adopt decoupled resource strategies above- and below-ground (Valverde-Barrantes et al., 2015; Blackman et al., 2016; Medeiros et al., 2017; Rodrigues et al., 2022), indicating that resource allocation varies among organs (Valverde-Barrantes et al., 2015). A multi-dimensional root trait syndrome has been observed, potentially driven by mycorrhizal associations, adaptive strategies to environmental constraints, or competitive interactions (Weemstra et al., 2016; Fort et al., 2017; Valverde-Barrantes et al., 2017; Wang et al., 2018; Bergmann et al., 2020; Carmona et al., 2021; Asefa et al., 2022). Therefore, more comprehensive investigations are required to elucidate the coordination of above- and below-ground traits in plant invasion.

Moso bamboo (*Phyllostachys edulis*) is a native giant “running” bamboo species widely distributed in subtropical China, comprising a significant proportion of bamboo forests (Song et al., 2016). Its rapid clonal reproduction enables it to expand and invade adjacent communities, leading to declines in plant diversity and alterations in soil fertility and microbial communities (Lima et al., 2012; Qin et al., 2017; Wu et al., 2018; Xu et al., 2020; Liu et al., 2021; Ouyang et al., 2022). As a native invasive species in subtropical regions, studying bamboo invasion provides valuable insights into the invasion process (Catford et al., 2019).

While previous studies have investigated changes in above- or below-ground traits in response to bamboo invasion, few have integrated both leaf and root traits or explored phenotypic integration comprehensively. Furthermore, existing studies often calculate the mean trait values of all species or dominant species in each sampling plot, neglecting the different contributions of various species to ecological processes and functions. Considering the importance of community-weighted mean trait value (CWM), which accounts for species abundance or important value (IV) in a community, a more comprehensive approach is necessary for plant invasion studies (Fried et al., 2019).

We employed a trait-based approach to address several critical questions: (1) Whether there are significant differences in functional traits between bamboo and broadleaf species, thus supporting the “phenotypic divergence hypothesis” or “phenotypic convergence hypothesis”? (2) Whether bamboo species exhibit stronger correlations of leaf and root traits, thus indicating higher phenotypic integration? (3) Whether above- and below-ground traits coordinate in response to bamboo invasion, thus dominated by a unidimensional plant economics spectrum or a multi-dimensional trait syndrome? Understanding these complex interactions will provide valuable insights into the mechanisms of bamboo invasion and contribute to sustainable development in subtropical evergreen broadleaf forest ecosystem.

## 2 Materials and methods

### 2.1 Study area

**Figure 1:**
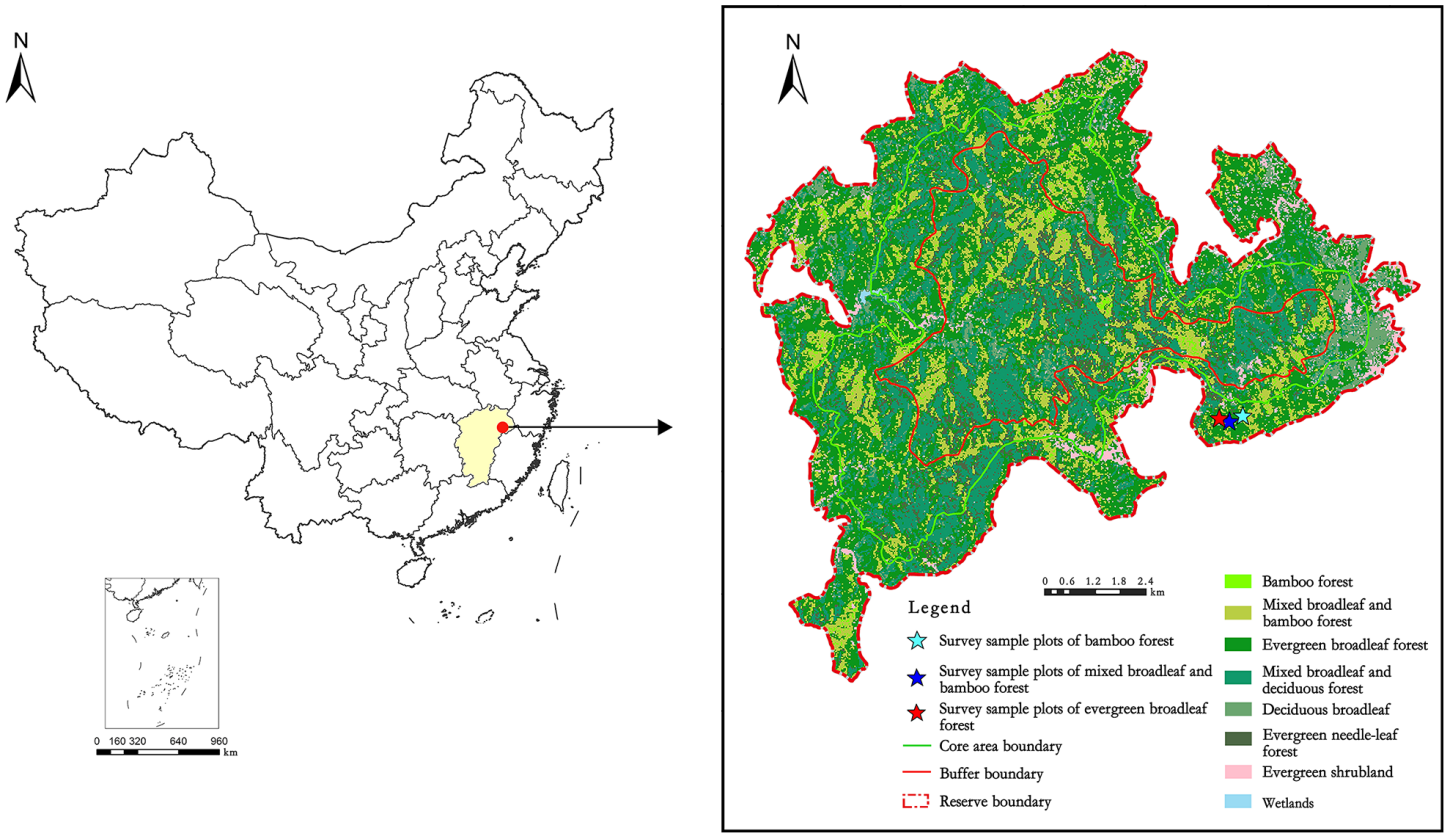
Location of the study area in Yangjifeng Nature Reserve in China.

The fieldwork was conducted in Yangjifeng National Nature Reserve with an approximate area of 10946 ha, located in Jiangxi Province, southeastern China, between 117°11′30″ - 117°28′40″ E longitude and 27°51′10″ - 28°02′20″ N latitude (**Figure 1**). The reserve serves as a significant ecological corridor, connecting various parts of the Wuyi Mountains. The area experiences a typical subtropical monsoon climate, with an average annual temperature of 14.4°C, annual precipitation of 2114 mm, relative humidity of 80%, and a frost-free period lasting 268 days (Zhang et al., 2023). The soil in the study site is classified as a Ferralsol in the FAO soil classification system (IUSS Working Group WRB, 2006), derived from granite, granite porphyry, and gneiss.

### 2.2 Experimental design and plant investigation

The study area within the nature reserve remains relatively free from anthropogenic disturbances. As a suitable species in subtropical China (Liu et al., 2021), bamboo is widely distributed in the study area, and rapidly invades into adjacent broadleaf forest. Three distinct forest types representing typical stages of bamboo invasion were identified: original evergreen broadleaf forest (EBF, uninvaded native forest), mixed broadleaf and bamboo forest (MF, transitional forest moderately invaded by bamboo), and pure bamboo forest (BF, completely invaded by bamboo).

In April 2017, three parallel transects with at least 10 m intervals along the bamboo invasion pathway from the bamboo forest to the evergreen broadleaf forest were set up, which were situated at elevations ranging from 687 m to 803 m. Three plots representing three forest types under relatively homogeneous environmental conditions (including slope, position, aspect and elevation) were established within each transect, spaced at least 10 m apart. The plot sizes were 10 m × 10 m for the bamboo forest given its relatively even distribution and 20 m × 20 m for the mixed forest and broadleaf forest. Therefore, a total of 9 plots (3 forest types × 3 replicates) were established. As the typical zonal forest, the evergreen broadleaf forest is generally dominated by *Castanopsis eyrie, Schima superba, Quercus glauca, Lithocarpus harlandii* and *Rhododendron latoucheae* (**Supplementary Table S1**). In the mixed broadleaf and bamboo forest, the canopy coverage per unit area of bamboo species was nearly equal to that of the broadleaf species, and is generally dominated by bamboo, *Castanopsis eyrie, Quercus glauca, Schima superba, Loropetalum chinense* (**Supplementary Table S1**). In the bamboo forest, the bamboo was the sole species in both the canopy and shrub layers. All trees and shrubs, including all bamboos and broadleaf species with a diameter at breast height (DBH) ≥ 5.0 cm, were measured, labeled, and recorded in terms of tree species, ages, tree height (height under the branch for bamboos), DBH, and crown width. The data collection was repeated in August 2018 (**Table 1**).

**Table 1:** Description of sampling plots in different forest types (mean ± SE).

Forest types	Plant species	Stand density (stems ha^-1^)	Average DBH (cm)	Average H (m)
BF	bamboo	4033 ± 47 a	8.4 ± 0.1 a	5.2 ± 0.5 a
EBF	broadleaf species	2030 ± 501 a	11.4 ± 2.0 a	7.5 ± 1.4 a
MF	bamboo	2044 ± 457 b	8.9 ± 0.5 a	6.1 ± 0.5 a
	broadleaf species	1147 ± 390 a	10.9 ± 0.7 a	7.0 ± 0.6 a

Shown are the mean values and associated standard errors. DBH, diameter at breast height; H, tree height for broadleaf species and height under branch for the bamboo; BF, the bamboo forest; MF, the mixed forest; EBF, the evergreen broadleaf forest. Different small letters in the same column represent significant differences in the same species between two forest types (P< 0.05).

### 2.3 Sampling

During the growing season in June 2017 and August 2018, leaf and root samples were collected from all plots. As a typical clonal species, bamboo can reproduce at different ages. Hence, the collection was done based on the classification of ages for bamboo and species for broadleaf trees. Bamboo age is represented as “du” to indicate the number of times it regenerates its leaves (Lin, 2012), and display the phenomenon of “on-year” production (Peng et al., 2020). Bamboo regenerates its leaves once a year in the first year, and once every two years thereafter, resulting in one “du” (I du) representing 1 year old, two “du” (II du) representing 2 and 3 years old, three “du” (III du) corresponding to 4 and 5 years old, and four “du” (IV du) corresponding to 6 and 7 years old (Lin, 2012). For this study, the focus was on bamboos of I du, II du, III du, and IV du, and three sample bamboos close to the average DBH of each age were selected from each plot.

Regarding broadleaf species, a total of 53 research objects from 25 species spanning 14 families in the evergreen broadleaf forests and the mixed forests were included (**Supplementary Table S2**). Some species were duplicated in different plots, and each was treated as an independent species resulting from different soil properties (Li et al., 2022). For each broadleaf species, three sample trees based on average DBH were selected. If there were less than three trees for a certain species in the plot, all available trees were sampled. The leaf samples were taken from the outer layer of the crown, and 5 mature fully expanded healthy leaves were randomly selected from each target individual in each direction to avoid any crown position effects. For root sampling, a soil block with a depth of 0-20 cm and an area of 10 cm × 10 cm was carefully excavated at a distance of 50 cm from the target individual in each direction, ensuring the branches of each fine root remained intact. All samples were stored at 4°C until they were transported to the laboratory for further analysis.

### 2.4 Trait measurement

A total of 34 functional traits were measured in our study. Leaf and fine root (root diameter< 2 mm) samples were scanned using an Epson V37 scanner (Seiko Epson Corporation, Japan). Three leaf thicknesses were measured at the top, middle, and bottom positions on the same side (avoiding primary veins) using a vernier caliper with an accuracy of 0.01 mm, and the average value was calculated as the leaf thickness (LT, mm). Leaf area (LA, cm^2^) was calculated using Image J software (National Institute of Health, Bethesda, Maryland, USA). Root length (RL, cm), root area (RA, cm^2^), root average diameter (RD, mm), and root volume (RV, cm^3^) were determined using WinRHIZO root-scanning software (Regent Instruments Inc., Ottawa, Canada).

An electronic balance with an accuracy of 0.0001 g was used to determine leaf fresh mass (LM_f_, g). Then, leaf samples were soaked in deionized water for 24 h in the dark to determine saturated fresh mass (LM_sf_, g). Subsequently, both leaf and root samples were oven-dried at 75°C to a constant mass, and leaf (LM_d_, g) and root dry mass (RM_d_, g) were determined. Specific leaf area (SLA, cm^2^ g^-1^), leaf dry matter content (LDMC, mg g^-1^), leaf tissue density (LTD, mg mm^-3^), leaf relative water content (LRWC, %), specific root length (SRL, m g^-1^), specific root area (SRA, cm^2^ g^-1^), root tissue density (RTD, mg cm^-3^), and root biomass (RB, kg m^-3^) were calculated using computational formulas (**Table 2**).

**Table 2:** Abbreviations, definitions (computational formulas) and units for 34 functional traits.

Organ	Traits	Abbreviations	Definitions (Computational formulas)	Units
Leaf	Leaf area	LA	Individual leaf area	cm^2^
	Specific leaf area	SLA	LA/LM_d_	cm^2^ g^-1^
	Leaf thickness	LT	Individual leaf thickness	mm
	Leaf tissue density	LTD	LM_d/_(LA × LT)	mg mm^-3^
	leaf dry matter content	LDMC	LM_d/_LM_sf_	mg g^-1^
	Leaf C concentration	LCC	Leaf C concentration per dry mass	mg g^-1^
	Leaf N concentration	LNC	Leaf N concentration per dry mass	mg g^-1^
	Leaf P concentration	LPC	Leaf P concentration per dry mass	mg g^-1^
	Leaf C/N	LCN	Leaf C concentration/N concentration	—
	Leaf C/P	LCP	Leaf C concentration/P concentration	—
	Leaf N/P	LNP	Leaf N concentration/P concentration	—
	Mass-based net photosynthetic rate	A_mass_	A_area_ × SLA	nmol g^-1^ s^-1^
	Mass-based dark respiration rate	R_mass_	R_area_ × SLA	nmol g^-1^ s^-1^
	Transpiration rate	E	—	mmol m^-2^ s^-1^
	Stomatal conductance	Gs	—	mmol m^-2^ s^-1^
	Intercellular carbon dioxide concentration	Ci	—	μmol mol ^−1^
	Stomatal limitation	Ls	(1- Ci)/air carbon dioxide concentrations	—
	Instantaneous carbon use efficiency	ICUE	(A_area_/(A_area_+ R_area_)) × 100%	%
	Photosynthetic N use efficiency	PNUE	(A_area_ × SLA)/LNC	µmol g^-1^ s^-1^
	Photosynthetic P use efficiency	PPUE	(A_area_ × SLA)/LPC	µmol mg^-1^ s^-1^
	Carboxylation efficiency	CE	A_area_/Ci	mol cm^-2^ s^-1^
	Leaf relative water content	LRWC	((LM_f_ - LM_d_)/(LM_sf_ - LM_d_)) ×100%	%
	Instantaneous water use efficiency	WUE	A_area_/E	µmol mmol^-1^
Root	Specific root length	SRL	RL/RM_d_	m g^-1^
	Specific root area	SRA	RA/RM_d_	cm^2^ g^-1^
	Root diameter	RD	Root average diameter	mm
	Root tissue density	RTD	RM_d_/RV	mg cm^-3^
	Root biomass	RB	RM_d_/soil sampling volume	kg m^-3^
	Root C concentration	RCC	Root C concentration per dry mass	mg g^-1^
	Root N concentration	RNC	Root N concentration per dry mass	mg g^-1^
	Root P concentration	RPC	Root P concentration per dry mass	mg g^-1^
	Root C/N	RCN	Root C concentration/N concentration	—
	Root C/P	RCP	Root C concentration/P concentration	—
	Root N/P	RNP	Root N concentration/P concentration	—

The chemical elements were determined after grinding the dried leaves and roots into a fine powder using a sample grinder and screening with a 1mm sieve. Leaf and root C and N concentrations per dry mass (LCC and LNC, RCC and RNC, respectively; mg g^-1^) were determined using a CHNS/O Elemental Analyzer (Vario EL III, Elementar, Germany). The leaf and root P concentrations per dry mass (LPC and RPC, respectively; mg g^-1^) were determined using a Continuous Flow Analytical System (SAN ++, Skalar, Holland) after H_2_SO_4_-HClO_4_ digestion. Leaf and root stoichiometric ratios were calculated.

Leaf photosynthetic parameters were measured using a portable LI-6400 photosynthesis system (LI-COR, Lincoln, Nebraska, USA) in June 2017. Measurements were conducted from 09:00 AM to 11:00 AM in the sunny and windless morning to avoid stomatal closure at midday. The measured parameters included area-based net photosynthetic rate (A_area_, μmol m^−2^ s^−1^), transpiration rate (E, mmol m^-2^ s^-1^), stomatal conductance (Gs, mmol m^-2^ s^-1^), and intercellular carbon dioxide (CO_2_) concentrations (Ci, μmol mol^−1^) of the healthy, fully expanded, upper leaves completely exposed to light in four directions from three representative individuals. Area-based dark respiration rate (R_area_, µmol m^-2^ s^-1^) was measured after at least 30 min of dark adaptation. Mass-based net photosynthetic rate (A_mass_, nmol g^-1^ s^-1^), mass-based dark respiration rate (R_mass_, nmol g^-1^ s^-1^), stomatal limitation (Ls), instantaneous carbon use efficiency (ICUE, %), photosynthetic N use efficiency (PNUE, µmol g^-1^ s^-1^), photosynthetic P use efficiency (PPUE, µmol mg^-1^ s^-1^), carboxylation efficiency (CE, mol cm^-2^ s^-1^), and instantaneous water use efficiency (WUE, µmol mmol^-1^) were calculated using computational formulas (**Table 2**).

### 2.5 Statistical analysis

The community-weighted mean trait value (CWM) was used to measure the functional composition of the community (Lodge et al., 2018; Sodhi et al., 2019). In this study, a community is referred to as a sampling plot. Plot-level mean trait values (CWM_plot_) were weighted by the Important Value (IV), manifesting the contribution of one species’ functional traits to the plot’s functional traits:


$${\text{CWM}}_{\text{plot}}=\sum_{\text{i}=1}^{\text{n}}{\text{IV}}_{\text{i}}\times {\text{ trait}}_{\text{i}}$$

where n is the number of species for broadleaf species and the number of age classes for bamboos in each plot; IV_i_ is the important value of species i for broadleaf and that of age i for bamboos, calculated as the mean of the relative abundance, relative significance, and relative height; trait_i_ is the trait value of species i for broadleaf and that of age i for bamboos.

Student’s t-test was used to compare the differences in leaf and root traits of the bamboo or broadleaf species between two different forest types and between the bamboo and broadleaf species in the mixed forests using SPSS 22 (SPSS Inc., Chicago, IL, USA). Graphs were created using Origin 2018 software (Origin Lab, Northampton, Massachusetts, USA).

Based on autocorrelation among the stoichiometry as well as photosynthetic traits, the C, N and P stoichiometric ratios were eliminated and only two photosynthetic traits (A_mass_ and R_mass_) were retained. Then, PCA analysis for the leaf and root was first performed to screen out the key traits, respectively (**Supplementary Figures S1A, B**). In the PCA analysis of leaf traits, PC1 explaining 41.6% of the variation indicated strong positive loadings on SLA, LNC and LPC, as well as negative loadings on LT (**Supplementary Figure S1A**). PC2 explaining 19.5% of the variation represented the strongest positive loading on LTD (**Supplementary Figure S1A**). In the PCA analysis of root traits, PC1 explaining 49.1% of the variation indicated strong positive loading on RTD and negative loadings on RPC and RNC (**Supplementary Figure S1B**). PC2 explaining 20.6% of the variation represented the strong positive loading on SRL and negative loading on RD (**Supplementary Figure S1B**). Hence, these five analogous trait pairs (LNC-RNC, LPC-RPC, SLA-SRL, LT-RD, and LTD-RTD) were selected as leaf and root key traits. Phenotypic integration was estimated using the key trait network (Messier et al., 2017; Michelaki et al., 2019; Rao et al., 2023; Sánchez-Bermejo et al., 2023), which included the number of significant key trait correlation pairs (Osunkoya et al., 2010, 2014; Luo et al., 2015; Zimmermann et al., 2016; Matesanz et al., 2021), and correlations of multiple traits between leaf and root traits. In our trait network, leaf and root traits are nodes, and trait-trait relationships are edges. Network estimation was conducted using the “qgraph” and “bootnet” packages in R software (v 4.2.1, R Core Development Team, 2022). Least absolute shrinkage and selection operator (LASSO) and extended Bayesian information criteria (EBIC) were applied for shrinking edges of a network, and the tuning parameter was set to 0.5 to sparsify the network (Bai et al., 2022). Centrality parameters, including closeness centrality, betweenness centrality, strength centrality, and Expected Influence, were calculated using the “qgraph” package in R (Epskamp et al., 2012). Considering the identification of hub traits (Kleyer et al., 2019; Matesanz et al., 2021; Rao et al., 2023), traits had the highest connectivity and Expected Influence were identified as hub traits, which play regulatory roles in plant phenotype (Rao et al., 2021). Pearson’s correlation analysis was conducted between the 10 leaf and root key traits to analyze the correlation using the “PerformanceAnalytics” package in R (Peterson et al., 2020). Multiple Factor Analysis (MFA), a multivariate ordination method, was used to analyze the correlation between two groups (i.e. leaf and root traits). RV (Robust variance), a between-group correlation coefficient, ranges from 0, representing complete uncorrelation in every variable between two groups, to 1, indicating perfect homotheticity in every variable between two groups (Baraloto et al., 2010). MFA was performed using the “FactoMineR” package in R (Lê et al., 2008).

Principal Component Analysis (PCA) was conducted on leaf, root, and integrated leaf and root traits to identify the resource strategies of the bamboo and broadleaf species using the “factoextra” package in R (Kassambara and Mundt, 2020). Permutational multivariate analysis of variance (PERMANOVA) were performed to detect the differences in different forest types and species using “adonis” function of “vegan” package (Oksanen et al., 2019). Visualization was done using the “ggplot2” and “corrplot” packages.

## 3 Results

### 3.1 Differences in leaf and root traits

**Figure 2:**
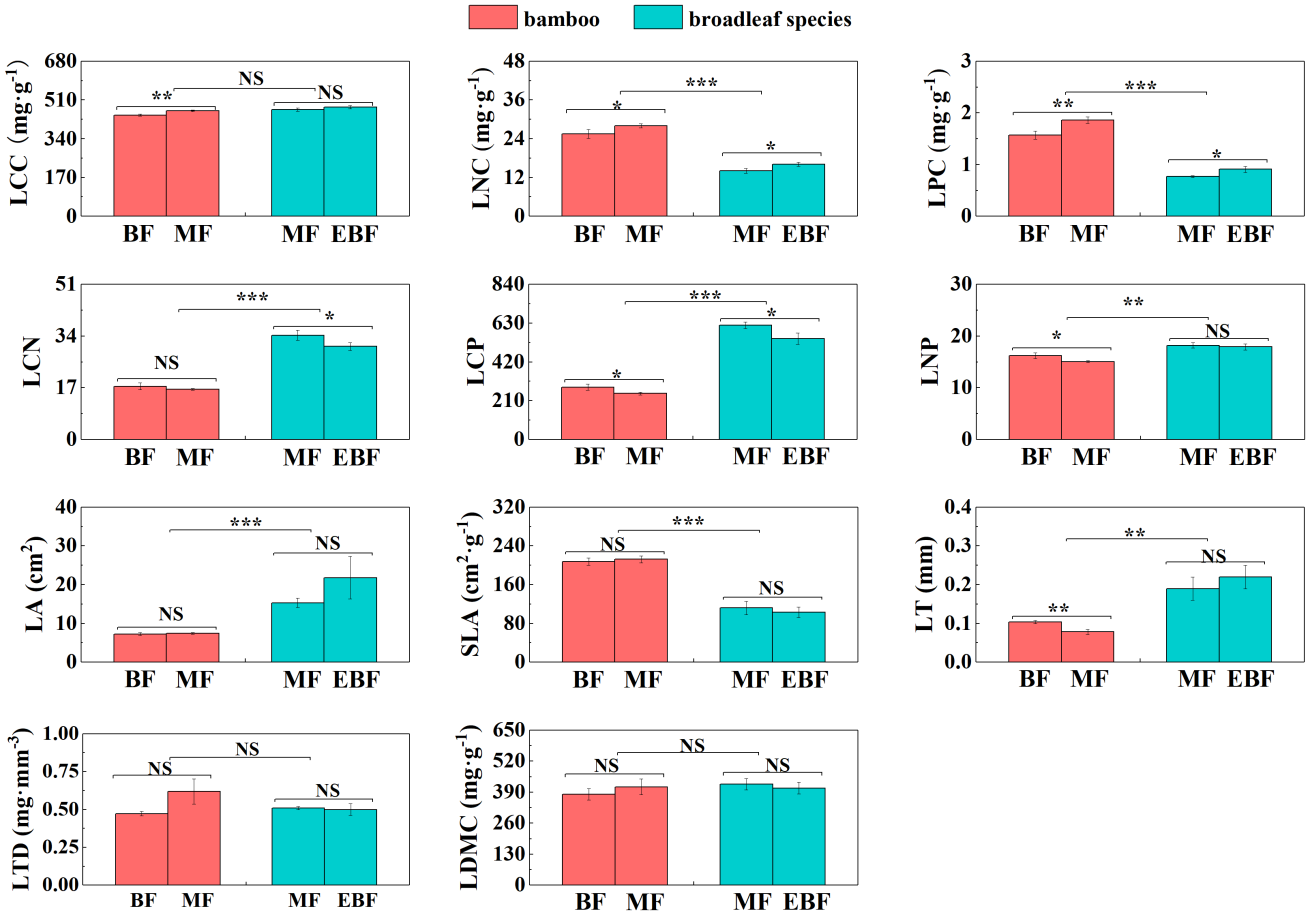
Differences in leaf C, N, and P stoichiometry and morphological traits (mean ± SD). BF, the bamboo forest; MF, the mixed forest; EBF, the evergreen broadleaf forest. ***< 0.001; **< 0.01; *< 0.05; NS indicated no significant difference. See **Table 2** for trait abbreviations.

**Figure 3:**
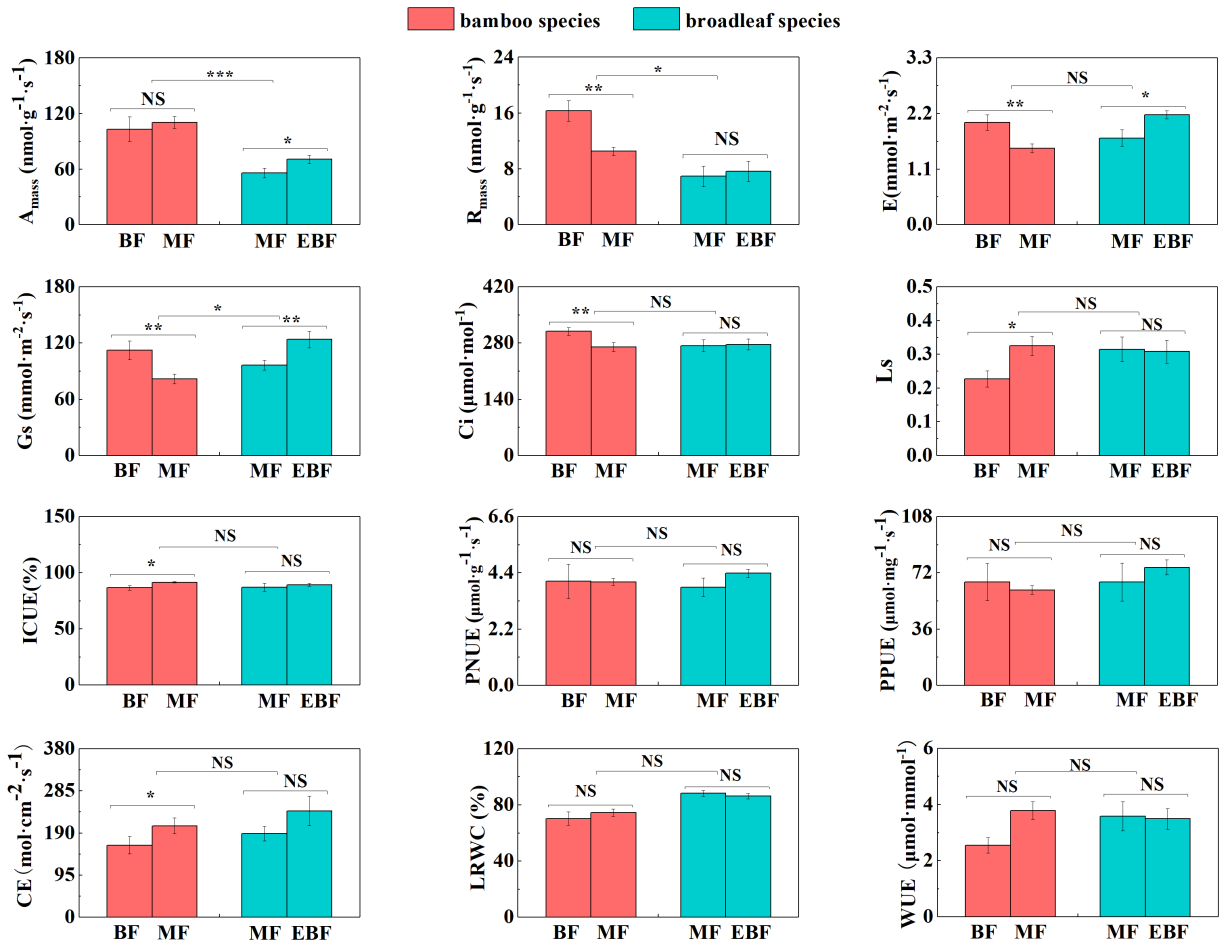
Differences in leaf photosynthetic traits (mean ± SD). BF, the bamboo forest; MF, the mixed forest; EBF, the evergreen broadleaf forest. ***< 0.001; **< 0.01; *< 0.05; NS indicated no significant difference. See **Table 2** for trait abbreviations.

Within the bamboo species, several leaf and root traits showed significant differences between the mixed forests and the bamboo forests. LCC, LNC, LPC, Ls, ICUE, and CE were significantly higher in the mixed forest (*P*< 0.05; **Figures 2**, **3**). Conversely, LCP, LNP, LT, R_mass_, E, Gs, and Ci were lower (*P*< 0.05; **Figures 2**, **3**).

**Figure 4:**
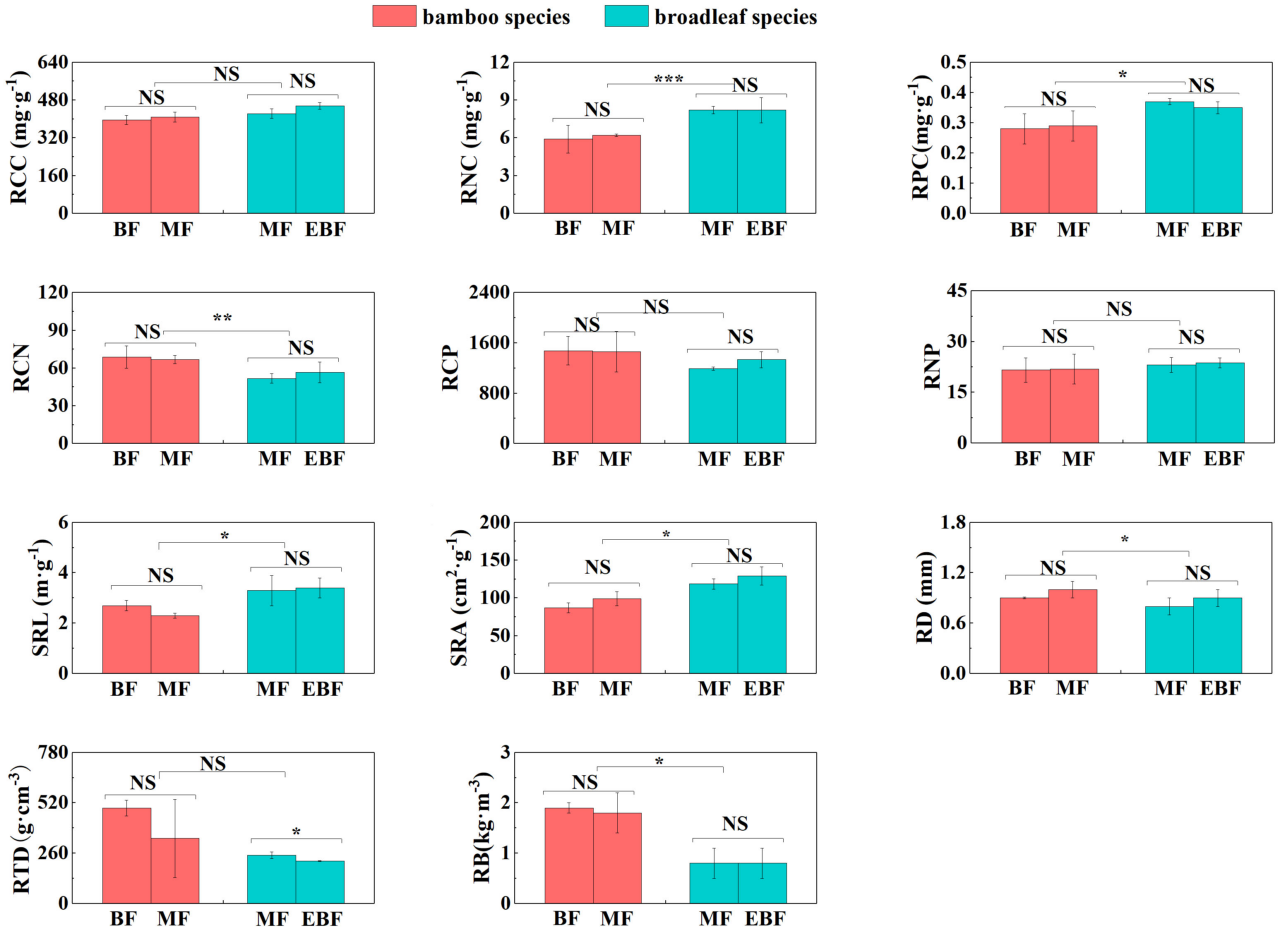
Differences in root traits (mean ± SD). BF, the bamboo forest; MF, the mixed forest; EBF, the evergreen broadleaf forest. ***< 0.001; **< 0.01; *< 0.05; NS indicated no significant difference. See **Table 2** for trait abbreviations.

For the broadleaf species, LNC, LPC, A_mass_, E, and Gs in the evergreen broadleaf forests were higher compared to the mixed forests (*P<* 0.05; **Figures 2**, **3**). Conversely, LCN, LCP, and RTD were significantly lower in the evergreen broadleaf forests (*P*< 0.05; **Figures 2**, **4**).

In the mixed forests after bamboo expansion, several leaf and root traits of bamboo species exhibited significant differences compared to the broadleaf species. Specifically, LNC, LPC, SLA, A_mass_, R_mass_, RCN, RD, and RB in the bamboo species were significantly higher (*P*< 0.05; **Supplementary Table S3**; **Figures 2**–**4**). On the other hand, LCN, LCP, LNP, LA, LT, Gs, RNC, RPC, SRL, and SRA were significantly lower (*P*< 0.05; **Supplementary Table S3**; **Figures 2**–**4**).

### 3.2 Correlations between leaf and root traits

**Figure 5:**
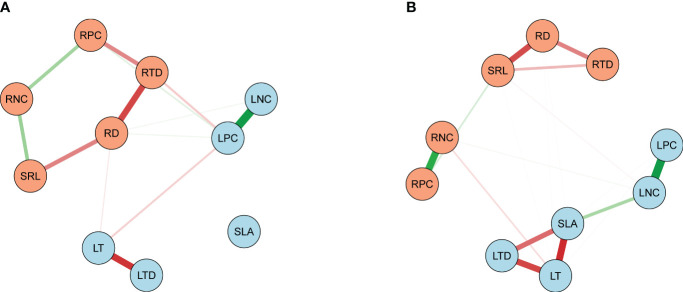
Traits’ correlation networks among the 10 leaf and root key traits. **(A)** Network analysis on the bamboo and **(B)** the broadleaf species. Light blue and light salmon nodes represent leaf and root traits, respectively. Green and red edges manifest the positive and negative correlations (*P*< 0.05), respectively. Thicker edges indicate stronger associations between two nodes. See **Table 2** for trait abbreviations.

**Figure 6:**
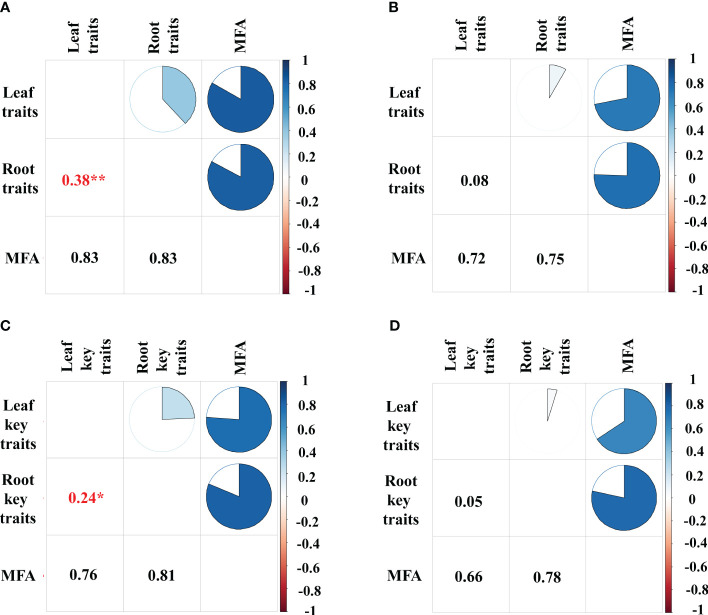
Multiple factor analysis between leaf and root traits. **(A)** MFA of 23 leaf traits and 11 root traits on the bamboo and **(B)** the broadleaf species; **(C)** MFA of 10 leaf and root key traits on the bamboo and **(D)** the broadleaf species. The gradient bar from dark blue to dark red indicates the RV (Robust variance) values ranging from 1 to -1. Numerical values in the graphs indicate the RV, and significant RV values are asterisked with red. The size of the sector in the pie chart represents the RV values. Blue in the pie chart represents positive RV values, and the darker the blue, the closer the RV values are to 1. **< 0.01; *< 0.05.

The bamboos demonstrated stronger correlations between leaf and root traits compared to the broadleaf species (**Figures 5**, **6**; **Supplementary Figures S2**, **S3**). Specifically, there were 16 pairs of significantly correlated leaf and root traits for the bamboo species (**Supplementary Figure S2**) and 11 pairs for the broadleaf species (**Supplementary Figure S3**) at a significance level of *P*< 0.05. The bamboos exhibited significant between-group correlations between leaf and root traits (*P*< 0.05; **Figures 6A, C**), while the broadleaf species did not show significant between-group correlations (*P* > 0.05; **Figures 6B, D**). These findings indicate that the bamboo species had higher phenotypic integration compared to the broadleaf species. Notably, among the traits studied, LNC was identified as the hub trait in our analysis (**Figure 5**; **Supplementary Figures S4**, **S5**).

### 3.3 Plant strategies

**Figure 7:**
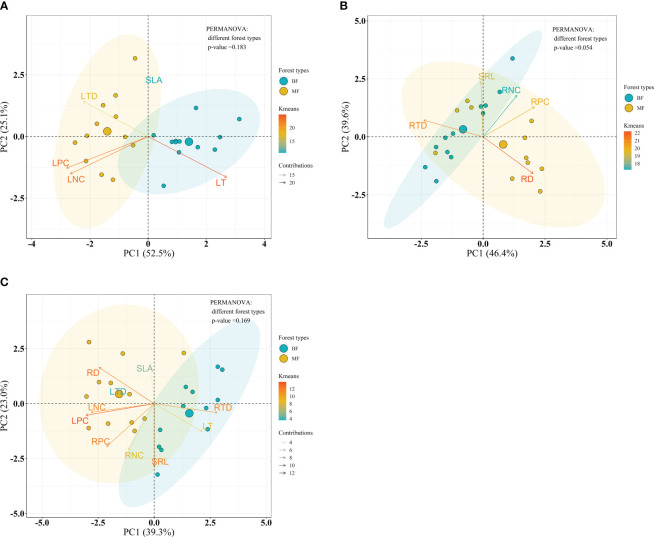
Bioplot of principal component analysis on key traits of bamboo species in different forests. **(A)** PCA on the leaf traits, **(B)** the root traits, and **(C)** the integrated leaf and root traits. BF, the bamboo forest; MF, the mixed forest. See **Table 2** for trait abbreviations.

**Figure 8:**
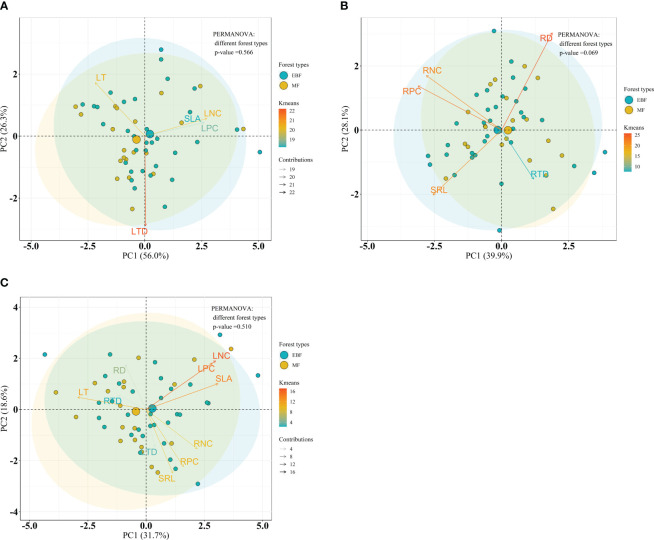
Bioplot of principal component analysis on key traits of broadleaf species in different forests. **(A)** PCA on the leaf traits, **(B)** the root traits, and **(C)** the integrated leaf and root traits. EBF, the evergreen broadleaf forest; MF, the mixed forest. See **Table 2** for trait abbreviations.

Considering the forest type, PERMANOVA analysis indicated no significant differences on leaf, root and the integrated leaf and root traits between forest types within the bamboo species (*P* = 0.183, *P* = 0.054 and *P* = 0.169, respectively) (**Figures 7A–C**), and within the broadleaf species (*P* = 0.566, *P* = 0.069 and *P* = 0.510, respectively) (**Figures 8A–C**).

**Figure 9:**
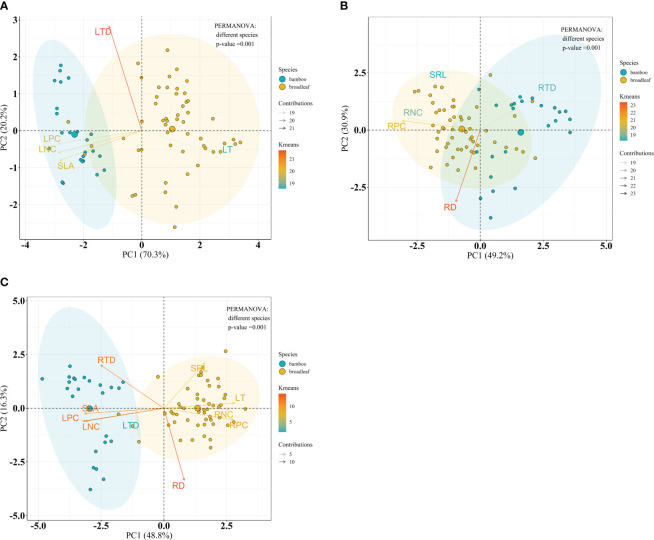
Bioplot of principal component analysis on key traits of bamboo and broadleaf species. **(A)** PCA on the leaf traits, **(B)** the root traits, and **(C)** the integrated leaf and root traits. See **Table 2** for trait abbreviations.

Considering the species, significant differences on leaf, root and the integrated leaf and root traits between bamboo and broadleaf species were detected (*P* = 0.001) (**Figures 9A–C**). In the leaf PCA analysis, the first and second principal components (PC1 and PC2) explained 70.3% and 20.2% of the total variance, respectively (**Figure 9A**). PC1 represented a variation along the leaf economics spectrum (LES) and was characterized by negative values, indicating high values of traits such as LNC, LPC, and SLA, which are associated with the acquisitive strategy of the bamboo species (**Supplementary Table S4**). On the other hand, positive values along PC1 were related to high LT, representing the conservative strategy of the broadleaf species (**Supplementary Table S4**). PC2 was primarily determined by LTD, showing a variation in leaf tissue density (**Supplementary Table S4**). Regarding the root traits, two principal components summarized the five traits and accounted for 80.1% of the total variance (**Figure 9B**). PC1, explaining 49.2% of the variance, was chiefly influenced by RPC, RNC, and RTD, representing a variation in nutrient traits and root tissue density along the root economics spectrum (RES) (**Supplementary Table S4**). Negative values along PC1, with high RPC and RNC, were associated with the acquisitive strategy of the broadleaf species, while positive values, with high RTD, indicated the conservative strategy of the bamboos (**Supplementary Table S4**). PC2, explaining 30.9% of the variance, was primarily loaded on RD and SRL, reflecting a variation in root thickness (**Supplementary Table S4**). The integration of all 10 leaf and root traits was expressed by three dimensions, accounting for 78.2% of the total variance (**Figure 9C**). The first dimension (48.8% variance) was mainly influenced by LPC, SLA, LNC, and LT, reflecting leaf economy (**Supplementary Table S4**). The second dimension (16.3% variance) was primarily loaded on RD and SRL, indicating root morphology (**Supplementary Table S4**). The third dimension (13.1% variance) was chiefly loaded on RPC and RNC, related to root nutrient traits (**Supplementary Table S4**).

## 4 Discussion

### 4.1 “Phenotypic convergence” or “phenotypic divergence”?

Our results indicated significant differences in most leaf and root key traits (i.e. LNC, LPC, LCN, LCP, LNP, SLA, LA, LT, A_mass_, R_mass_, Gs, RNC, RPC, RCN, SRL, SRA, RD, and RB) between bamboo and broadleaf species, providing strong support for the “phenotypic divergence hypothesis”.

It is well recognized that differences in leaf and root traits between plant life forms (e.g., bamboo and broadleaf species belonging to monocotyledons of Poaceae and woody plants, respectively) lead to variations in resource demand and utilization, such as light, water, and nutrients. SLA, which characterizes a plant’s ability to capture light and assimilate CO_2_, has been shown to distinguish invasive and native species effectively. The higher SLA with thinner leaves in bamboo species enhances CO_2_ assimilation capacity at a relatively low cost, while lower Gs facilitates more effective stomatal control, contributing to their competitive advantage over broadleaf species. Notably, bamboo leaf anatomy was found to differ from typical “kranz anatomy” observed in other gramineous plants with C4 photosynthetic pathway but resembled that of broadleaf species with C3 photosynthetic pathway. However, bamboo species displayed significantly higher A_mass_ and R_mass_ compared to broadleaf species, indicating superior assimilate production capacity and photosynthetic efficiency, consistent with findings from other bamboo species (*Chusquea ramosissima* and *Chusquea tenella*) (Montti et al., 2014). Besides, bamboo can benefit from its competitive advantages not only in full light environment, but also in light-limited environment (Wang et al., 2016). Therefore, it can hinder broadleaf species from capturing light resources, forming a shadowing effect and thus inhibiting the growth and regeneration of broadleaf species. Bamboo species also exhibited higher leaf N and P concentrations, photosynthetic, and respiration rates than broadleaf species, similar to what has been documented for most invasive species (Heberling and Mason, 2018; Mathakutha et al., 2019; Díaz de León Guerrero et al., 2020; Palma et al., 2021; Wang et al., 2021; Montesinos, 2022). These traits demonstrate elevated leaf nutrient status and photosynthetic potential, supporting their competitive advantage. However, bamboo species showed lower root N and P concentrations, as suggested in previous research (Ning et al., 2017), where bamboo species as monocotyledons of Poaceae are typically characterized by lower root N and P but higher root C:N ratio.

The contrasting N and P concentrations between leaves and roots may result from the differences in organ structure and function during the growing season (Minden and Kleyer, 2014). Vigorous photosynthesis in the growing season without water limitation requires a large amount of N and P for the production of photosynthetic proteins and chlorophyll, as well as for ATP synthesis and enzyme catalysis. As a result, bamboo species allocate more N and P to leaves than to roots in comparison to broadleaf species, suggesting fast above-ground growth to gather more light in the understory. As a shade-tolerant species (Wang et al., 2016), bamboos species have low light compensation point, beneficial to capturing low light. Therefore, it seems that under the mixed canopy, bamboo species are considered to be good competitors in low-light conditions (Wang et al., 2016). Roots, on the other hand, encounter more complex soil habitats, leading to greater variability and uncertainty in root traits compared to leaf traits. Thicker roots with lower specific root length and specific root area in bamboo species facilitate penetration of harder soils (Mathakutha et al., 2019), reduce hydraulic failure (Lozano et al., 2020; Asefa et al., 2022), and acquire more soil resources via fungal extraradical hyphae (Valverde-Barrantes et al., 2017; de la Riva et al., 2018). This trait divergence allows bamboo species to access vacant niches in recipient communities, potentially facilitating their invasion into broadleaf forests. Therefore, establishment of broadleaf species with key traits similar to bamboo species may confer the resistance of bamboo invasion and maintain subtropical forest ecosystem function.

However, some traits may fluctuate during the growing season, particularly if resources also fluctuate (Wang et al., 2022). Additionally, it was reported that trait divergence was habitat-dependent, strongly influencing by environmental conditions (Tecco et al., 2010; Luo et al., 2015). As a result, the “phenotypic divergence hypothesis” may not work in stressful environments.

### 4.2 Higher phenotypic integration for the bamboos?

In our study, we estimated phenotypic integration using a trait network (Messier et al., 2017; Michelaki et al., 2019; Rao et al., 2023; Sánchez-Bermejo et al., 2023) and individual trait-pair correlations between leaf and root traits (Osunkoya et al., 2010, 2014; Luo et al., 2015; Zimmermann et al., 2016; Matesanz et al., 2021). The results showed stronger trait correlations and more significant correlations between individual leaf and root traits in bamboo species compared to broadleaf species, in conformity with previous studies on invasive vines (Osunkoya et al., 2010, 2014) and *Acer pseudoplatanus* (Shouman et al., 2020). These findings indicated higher phenotypic integration in bamboo species. Highly integrated phenotypes in plants allow for better adaptation to environmental changes, enabling efficient acquisition of resources and response to environmental stresses (Gianoli and Palacio-López, 2009; Luo et al., 2015). Consequently, higher phenotypic integration in bamboos contributes to their superior performance and facilitates invasion into broadleaf forests. Specifically, LNC characterized by the highest Expected Influence and connectivity (Rao et al., 2023), was identified as the hub trait, further supporting the concept of phenotypic integration. Our findings emphasized the importance of phenotypic integration in regulating bamboo invasion. Broadleaf species with higher trait correlation can be selected to enhance invasion resistance.

Interestingly, while previous literature emphasized the roles of SLA and LNC as primary traits coordinating with others in the leaf economic spectrum (Wright et al., 2004), our trait network did not emphasize SLA. This discrepancy may be due to weak correlations between SLA and other traits in bamboo species, as identified by Messier et al. (2017). Moreover, our study revealed differences in the relationship between leaf and root traits among bamboo and broadleaf species, indicating that plant phylogeny, including evolution and taxonomy, can have a greater impact on functional traits than environmental factors (Figueroa and Armesto, 2001; Wang et al., 2018; Liu et al., 2019; Wang et al., 2024). The weak correlation between leaf and root traits in broadleaf species suggests that broadleaf species integrate traits in various ways to improve their fitness in response to bamboo invasion.

### 4.3 Does a unidimensional plant economic spectrum or a multi-dimensional trait syndrome dominate above- and below-ground strategies?

Our results indicated strong trade-off among leaf traits, representing a unidimensional leaf economic spectrum (Wright et al., 2004). The leaf “conservation” gradient of the LES, which describes a trade-off between leaf traits associated with a “slow” to “fast” return on resource investment, is evident in bamboo species with higher SLA, LNC, LPC, A_mass_, and R_mass_, and lower LA and LT. This acquisitive strategy enables bamboo species to effectively capture light and absorb nutrients and water resources, providing them with a competitive edge over broadleaf species. In contrast, the evergreen broadleaf species in our study, with lower SLA, larger leaf area, and thicker leaves, display a “slow investment - return” strategy, investing more biomass in leaf tissue toughness and stem growth.

However, our study also revealed a two-dimensional root trait syndrome, indicating a trade-off between root traits in bamboo and broadleaf species. The significantly negative correlation between RNC and RTD represents a “conservation” gradient, indicating the classical root economics spectrum characterized by root acquisition-conservation trade-off. Bamboo species exhibit lower RNC and RPC and higher RTD, representing a “slow conservative” strategy, whereas broadleaf species displayed a “fast acquisitive” strategy with higher RNC and RPC and lower RTD, indicating that bamboo species develop acquisitive leaf traits for photosynthesis maximization and conservative root traits for water and nutrient storage. This trade-off between above- and below-ground strategies supports the notion that above-ground resource utilization strategies may not necessarily correspond to below-ground strategies. It was documented that root “fast-slow” trade-off was influenced by soil nutrient limitation (Li et al., 2019). In our study, the average total N, total P and available P concentrations in bamboo rhizosphere soil were lower than those in broadleaf rhizosphere soil (our unpublished data). Besides, soil in subtropical China is deficient in P (Tian et al., 2010). Hence, bamboo species adopt “slow conservative” strategy to conserve water and nutrient resources in poor soil conditions. The strong negative correlation between SRL and RD indicates the existence of a “collaboration” gradient, ranging from a “do-it-yourself” strategy associated with high SRL to an “outsourcing” strategy related to thick roots (Bergmann et al., 2020; Weigelt et al., 2021). Fagaceae species, dominant in the broadleaf and mixed forests in our study, are characterized by ectomycorrhizal (EcM) (Kong et al., 2014). Accordingly, it seems that broadleaf species with higher SRL more likely colonized by EcM fungi are apt to occupy the extreme of “do-it-yourself”, while bamboo species with thicker roots preferred by arbuscular mycorrhizal (AM) fungi are inclined to be “outsourcing” (Laliberté, 2017; Bergmann et al., 2020). Ample evidence suggests that EcM species characterized by high SRL probably stem not only from the nature of the EcM symbiosis depending less on the cortex area of their roots, but also from their more recent evolution, on account of young species associated with thinner roots evolutionarily (Brundrett, 2002; Valverde-Barrantes et al., 2017; Bergmann et al., 2020). Therefore, the phylogenetic differences between bamboo and broadleaf species probably further support the observed root trait syndrome, and suggest that bamboo species colonize a distinct niche in forests not occupied, at least in part, by broadleaf species.

On the whole, our findings suggest that multiple resource utilization dimensions should be considered for understanding plant ecological strategies, with a multi-dimensional trait syndrome dominating above- and below-ground strategies, rather than a unidimensional plant economic spectrum.

## 5 Conclusion

In conclusion, our study investigated the leaf and root traits of bamboo and broadleaf species, aiming to understand whether phenotypic convergence or phenotypic divergence occurred between these two groups. Our results revealed significant differences in most leaf and root key traits, including LNC, LPC, LCN, LCP, LNP, SLA, LA, LT, A_mass_, R_mass_, Gs, RNC, RPC, RCN, SRL, SRA, RD, and RB. These findings strongly supported the “phenotypic divergence hypothesis” rather than the “phenotypic convergence hypothesis”.

Furthermore, we explored the level of phenotypic integration between leaf and root traits in both bamboo and broadleaf species. Bamboos exhibited stronger trait correlations and more significant correlations between individual leaf and root traits, indicating higher phenotypic integration and providing them with competitive advantages. Interestingly, we identified LNC as the hub trait characterized by the highest Expected Influence in the trait network.

However, we found that above- and below-ground traits were not coordinated, and a multi-dimensional trait syndrome was observed. It was characterized by LNC, LPC, SLA and LT contributing to leaf “conservation” gradient, RNC and RTD contributing to root “conservation” gradient, as well as SRL and RD contributing to “collaboration” gradient. Our results demonstrated a unidimensional leaf economic spectrum with bamboo species showing acquisitive leaf strategies, characterized by higher LNC, LPC, and SLA, occupying the “fast investment - return” extreme, while broadleaf species exhibited conservative leaf strategies with thicker leaves at the “slow investment - return” extreme. Notably, this unidimensional LES was counterbalanced by a two-dimensional root trait syndrome. Bamboos displayed “slow conservative” root strategies with higher RTD, compensating for their acquisitive leaf traits. In contrast, broadleaf species exhibited “fast acquisitive” root strategies with higher RNC and RPC, counteracting their conservative leaf strategies. This trade-off between above- and below-ground strategies provided additional insights into plant resource allocation, revealing a “conservation” gradient. Another “collaboration” gradient probably ranged from a broadleaf “do-it-yourself” strategy associated with higher SRL to a bamboo “outsourcing” strategy related to thicker roots.

We predict that bamboos are more efficient understory species able to coexist with broadleaf species, but when invasive bamboos dominate the regenerative niches in the sense that bamboos are better competitors for light in the understory, making the regeneration of broadleaf species difficult. Thus, if there is not a catastrophic perturbation, with the passing of time when adult broadleaf species die, the mixed forests will be converted into bamboo forests. Therefore, in order to efficiently prevent bamboo invasion and maintain the sustainable development of evergreen broadleaf forests, plant functional traits should be taken into account. Effective controlling measures should be adopted to screen out key traits indicating bamboo superior to broadleaf species, or select broadleaf species based on trait similarity characterized by high phenotypic integration to coexist with bamboo species. Overall, our findings deepen the understanding of bamboo invasion into adjacent evergreen broadleaf forests and provide scientific guidance for promoting the sustainable development of subtropical forest ecosystems. For future research, we will integrate more above- and below-ground traits and anatomically identify mycorrhiza types of broadleaf species to acquire a comprehensive understanding of plant ecological strategies and their ecological implications.

## Data availability statement

The original contributions presented in the study are included in the article/**Supplementary Material**. Further inquiries can be directed to the corresponding authors.

## Author contributions

HY: Conceptualization, Data curation, Formal analysis, Funding acquisition, Methodology, Software, Visualization, Writing – original draft, Writing – review & editing. XL: Data curation, Investigation, Writing – review & editing. JP: Supervision, Writing – review & editing. JS: Supervision, Writing – review & editing. CX: Data curation, Investigation, Writing – review & editing. YZ: Investigation, Software, Writing – review & editing. ZX: Data curation, Investigation, Writing – review & editing. CL: Data curation, Investigation, Writing – review & editing. ZM: Data curation, Investigation, Writing – review & editing. DC: Funding acquisition, Supervision, Writing – review & editing. QZ: Funding acquisition, Methodology, Supervision, Writing – review & editing.
