# Supplementary material for: Trait divergence and opposite above- and below-ground strategies facilitate moso bamboo invasion into subtropical evergreen broadleaf forest
Source: Front Plant Sci. 2024 Jul 19;15:1410372. doi: 10.3389/fpls.2024.1410372 (PMC11294163; doi:10.3389/fpls.2024.1410372)
Supplement: Supplementary file 1 [file DataSheet_1.docx]

Supplementary material

Trait divergence and opposite above- and below-ground strategies facilitate moso bamboo invasion into subtropical evergreen broadleaf forest

Hua Yu, Xingui Le, Josep Peñuelas, Jordi Sardans, Chaobin Xu, Yuxing Zou, Xue Zhang, Conghui Li, Zhenwei Mao, Dongliang Cheng, Quanlin Zhong*

*** Correspondence:** Quanlin Zhong: [qlzhong@126.com](mailto:qlzhong@126.com)

# Supplementary Tables

**Table S1** Stand composition for the evergreen broadleaf forests and the mixed forests

| Forest types | Species | IV (%) | Forest type | Species | IV (%) |
| --- | --- | --- | --- | --- | --- |
| EBF | *Castanopsis eyrei* | 17.1% | MF | *Castanopsis eyrei* | 10.6% |
|  | *Schima superba* | 14.1% |  | *Quercus glauca* | 6.2% |
|  | *Quercus glauca* | 13.7% |  | *Schima superba* | 5.6% |
|  | *Lithocarpus harlandii* | 9.9% |  | *Loropetalum chinense* | 4.6% |
|  | *Rhododendron delavayi* | 8.9% |  | *Castanopsis fargesii* | 4.4% |
|  | *Alniphyllum fortunei* | 5.0% |  | *Rhododendron delavayi* | 3.8% |
|  | *Elaeocarpus decipiens* | 4.0% |  | *Eurya muricata* | 3.5% |
|  | *Eurya muricata* | 3.7% |  | *Chimonanthus praecox* | 2.9% |
|  | *Castanopsis tibetana* | 3.3% |  | *Camellia oleifera* | 2.0% |
|  | *Choerospondias axillaris* | 3.2% |  | *Alniphyllum fortunei* | 1.7% |
|  | *Ilex chinensis* | 2.7% |  | *Toxicodendron succedaneum* | 1.5% |
|  | *Loropetalum chinense* | 2.2% |  | *Litsea cubeba* | 1.0% |
|  | *Litsea cubeba* | 2.2% |  | *Clausena lansium* | 0.8% |
|  | *Ternstroemia gymnanthera* | 1.8% |  | *Phyllostachys edulis* | 51.2% |
|  | *Litsea coreana* | 1.6% |  |  |  |
|  | *Toxicodendron succedaneum* | 1.5% |  |  |  |
|  | *Dendropanax dentiger* | 1.4% |  |  |  |
|  | *Prunus dielsiana* | 1.4% |  |  |  |
|  | *Lithocarpus laber* | 0.6% |  |  |  |
|  | *Chimonanthus praecox* | 0.6% |  |  |  |
|  | *Machilus thunbergii* | 0.5% |  |  |  |
|  | *Albizia kalkora* | 0.5% |  |  |  |

EBF: the evergreen broadleaf forests; MF: the mixed broadleaf and bamboo forests; IV: the important value.

**Table S2** The sampling evergreen broadleaf species.

| Forest types | Plots | Species | Families | Sampling numbers |  |
| --- | --- | --- | --- | --- | --- |
| EBF | EBF1 | *Castanopsis eyrei* | Fagaceae | 3 | 11 families  12 species |
|  |  | *Quercus glauca* | Fagaceae | 3 |  |
|  |  | *Eurya muricata* | Pentaphylacaceae | 3 |  |
|  |  | *Schima superba* | Theaceae | 3 |  |
|  |  | *Elaeocarpus decipiens* | Elaeocarpaceae | 3 |  |
|  |  | *Prunus dielsiana* | Rosaceae | 3 |  |
|  |  | *Toxicodendron succedaneum* | Anacardiaceae | 3 |  |
|  |  | *Alniphyllum fortunei* | Styracaceae | 3 |  |
|  |  | *Dendropanax dentiger* | Araliaceae | 3 |  |
|  |  | *Rhododendron delavayi* | Ericaceae | 3 |  |
|  |  | *Chimonanthus praecox* | Calycanthaceae | 3 |  |
|  |  | *Machilus thunbergii* | Lauraceae | 2 |  |
|  | EBF2 | *Castanopsis eyrei* | Fagaceae | 3 | 9 families  11 species |
|  |  | *Quercus glauca* | Fagaceae | 3 |  |
|  |  | *Lithocarpus harlandii* | Fagaceae | 3 |  |
|  |  | *Schima superba* | Theaceae | 3 |  |
|  |  | *Rhododendron delavayi* | Ericaceae | 3 |  |
|  |  | *Ilex chinensis* | Aquifoliaceae | 3 |  |
|  |  | *Loropetalum chinense* | Hamamelidaceae | 3 |  |
|  |  | *Choerospondias axillaris* | Anacardiaceae | 3 |  |
|  |  | *Eurya muricata* | Pentaphylacaceae | 3 |  |
|  |  | *Alniphyllum fortunei* | Styracaceae | 3 |  |
|  |  | *Ternstroemia gymnanthera* | Pentaphylacaceae | 3 |  |
|  | EBF3 | *Castanopsis eyrei* | Fagaceae | 3 | 5 families  10 species |
|  |  | *Castanopsis tibetana* | Fagaceae | 3 |  |
|  |  | *Lithocarpus laber* | Fagaceae | 3 |  |
|  |  | *Lithocarpus harlandii* | Fagaceae | 3 |  |
|  |  | *Albizia kalkora* | Fabaceae | 3 |  |
|  |  | *Schima superba* | Theaceae | 3 |  |
|  |  | *Rhododendron delavayi* | Ericaceae | 3 |  |
|  |  | *Eurya muricata* | Pentaphylacaceae | 3 |  |
|  |  | *Litsea coreana* | Lauraceae | 3 |  |
|  |  | *Litsea cubeba* | Lauraceae | 3 |  |
| MF | MF1 | *Castanopsis eyrei* | Fagaceae | 3 | 5 families  7 species |
|  |  | *Quercus glauca* | Fagaceae | 3 |  |
|  |  | *Schima superba* | Theaceae | 3 |  |
|  |  | *Camellia oleifera* | Theaceae | 3 |  |
|  |  | *Eurya muricata* | Pentaphylacaceae | 2 |  |
|  |  | *Rhododendron delavayi* | Ericaceae | 2 |  |
|  |  | *Loropetalum chinense* | Hamamelidaceae | 2 |  |
|  | MF2 | *Castanopsis eyrei* | Fagaceae | 3 | 5 families  7 species |
|  |  | *Quercus glauca* | Fagaceae | 3 |  |
|  |  | *Castanopsis fargesii* | Fagaceae | 3 |  |
|  |  | *Schima superba* | Theaceae | 3 |  |
|  |  | *Litsea cubeba* | Lauraceae | 3 |  |
|  |  | *Toxicodendron succedaneum* | Anacardiaceae | 2 |  |
|  |  | *Alniphyllum fortunei* | Styracaceae | 1 |  |
|  | MF3 | *Castanopsis eyrei* | Fagaceae | 3 | 4 families  6 species |
|  |  | *Castanopsis fargesii* | Fagaceae | 3 |  |
|  |  | *Quercus glauca* | Fagaceae | 3 |  |
|  |  | *Schima superba* | Theaceae | 3 |  |
|  |  | *Chimonanthus praecox* | Calycanthaceae | 2 |  |
|  |  | *Clausena lansium* | Rutaceae | 1 |  |
| Total | | 25 species | 14 families | 53 research objects  149 samples | |

EBF: the evergreen broadleaf forests; MF: the mixed broadleaf and bamboo forests.

**Table S3** Significant differences in traits between bamboo and broadleaf species in the mixed forest (mean ± SD)

| Species | LNC (mg∙g^-1^) | LPC (mg∙g^-1^) | LCN | LCP | LNP | LA (cm^2^) | SLA ( cm^2^∙g^-1^) | LT (mm) | A_mass_ (nmol∙g^-1^∙s^-1^) |
| --- | --- | --- | --- | --- | --- | --- | --- | --- | --- |
| bamboo | 28.1±0.7a | 1.9±0.06a | 16.5±0.3b | 249.1±7.6b | 15.1±0.2b | 7.5±0.2b | 212.3±7.0a | 0.1±0.4b | 110.4±6.5a |
| broadleaf | 14.1±0.8b | 0.8±0.02b | 34.3±1.7a | 619.0±17.8a | 18.2±0.5a | 15.3±1.2a | 112.0±14.0b | 0.8±0.3a | 55.8±5.3b |
| Species | R_mass_ (nmol∙g^-1^∙s^-1^) | Gs (mmol∙m^-2^∙s^-1^) | RNC (mg∙g^-1^) | RPC (mg∙g^-1^) | RCN | SRL (m∙g^-1^) | SRA (cm^2^∙g^-1^) | RD (mm) | RB (kg∙m^-3^) |
| bamboo | 10.5±0.6a | 81.8±5.1b | 6.2±0.1b | 0.29±0.05b | 66.8±3.2a | 2.3±0.1b | 99.1±9.1b | 1.0±0.1a | 1.8±0.4a |
| broadleaf | 6.9±1.5b | 96.5±5.3a | 8.2±0.3a | 0.37±0.01a | 51.8±3.8b | 3.3±0.6a | 118.6±6.7a | 0.8±0.1b | 0.8±0.3b |

Shown are the mean values and associated standard deviation. Different small letters in the same column indicate significant differences between the bamboo species and the broadleaf species in the mixed forests (*P* < 0.05). See Table 2 for trait abbreviations.

**Table S4** Loadings of the first three principal components for PCA analysis on key traits of bamboo and broadleaf species

| Organ | Traits | Loadings of the first three principal components | | |
| --- | --- | --- | --- | --- |
|  |  | PC1 | PC2 | PC3 |
| Leaf | LNC | -0.696 | -0.191 | -0.430 |
|  | LPC | -0.699 | -0.113 | -0.486 |
|  | SLA | -0.628 | -0.260 | 0.402 |
|  | LT | 0.476 | -0.209 | -0.621 |
|  | LTD | -0.195 | 0.916 | -0.178 |
| Root | RNC | -0.618 | 0.128 | -0.508 |
|  | RPC | -0.677 | 0.097 | -0.313 |
|  | SRL | -0.356 | 0.521 | 0.582 |
|  | RD | -0.182 | -0.744 | -0.007 |
|  | RTD | 0.553 | 0.386 | -0.456 |
| Whole plant | LNC | -0.593 | -0.131 | -0.192 |
|  | LPC | -0.605 | -0.131 | -0.169 |
|  | SLA | -0.597 | -0.053 | -0.161 |
|  | LT | 0.554 | 0.054 | 0.439 |
|  | LTD | -0.137 | -0.118 | -0.388 |
|  | RNC | 0.329 | -0.008 | -0.623 |
|  | RPC | 0.346 | -0.116 | -0.630 |
|  | SRL | 0.206 | 0.559 | -0.396 |
|  | RD | 0.102 | -0.728 | 0.106 |
|  | RTD | -0.316 | 0.437 | 0.183 |

PC1: the first principal component; PC2: the second principal component; PC3: the third principal component. See Table 2 for trait abbreviations.

# Supplementary Figures

**
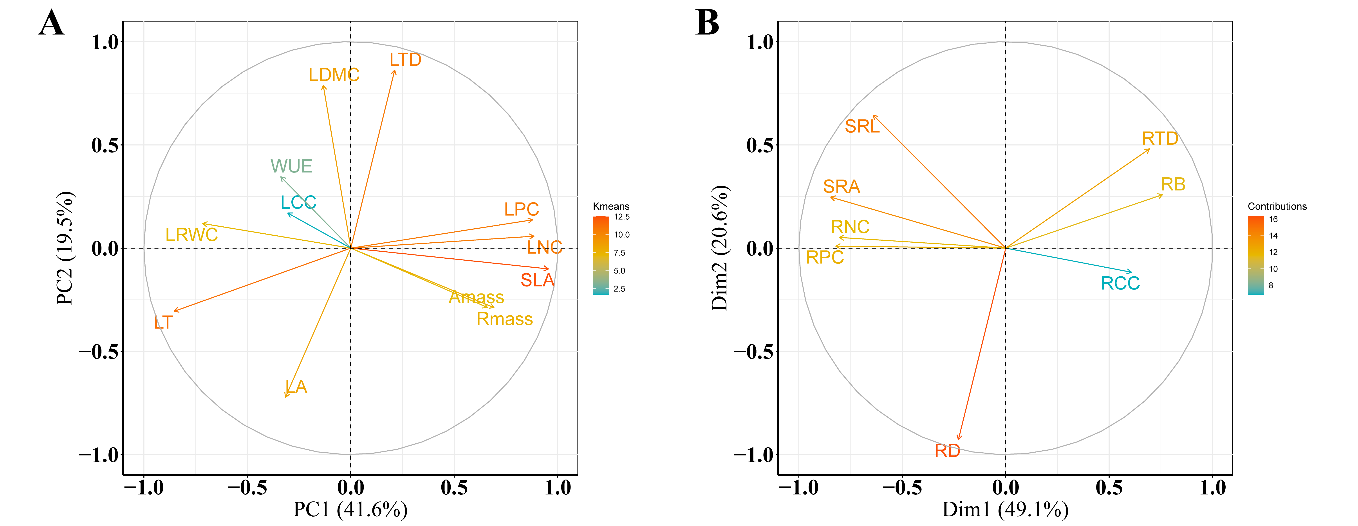
**

**Figure S1.** Bioplot of principal component analysis on traits of bamboo and broadleaf species. (A) PCA on the leaf traits and (B) the root traits. See Table 2 for trait abbreviations.

**
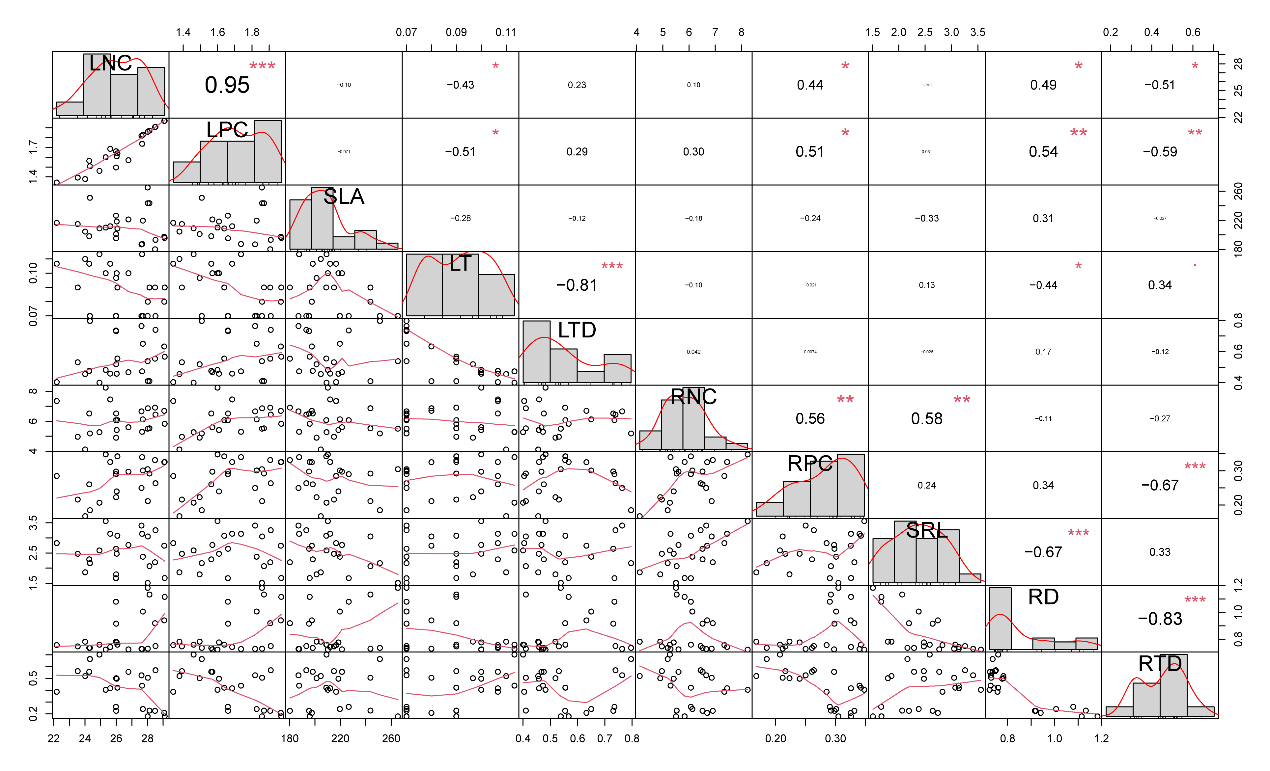
**

**Figure S2.** Correlation analysis on leaf and root key traits of the bamboos. *** < 0.001; ** < 0.01; * < 0.05. See Table 2 for trait abbreviations.

**
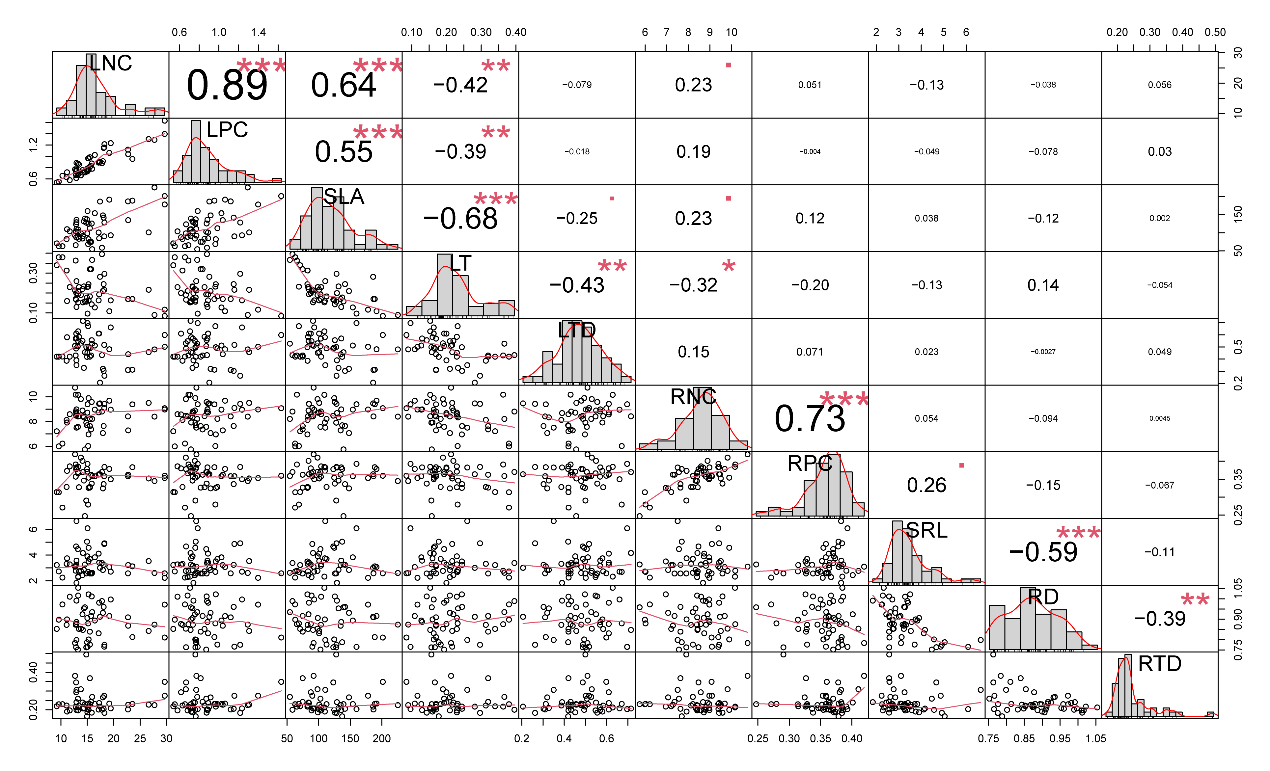
**

**Figure S3.** Correlation analysis on leaf and root key traits of the broadleaf species. *** < 0.001; ** < 0.01; * < 0.05. See Table 2 for trait abbreviations.


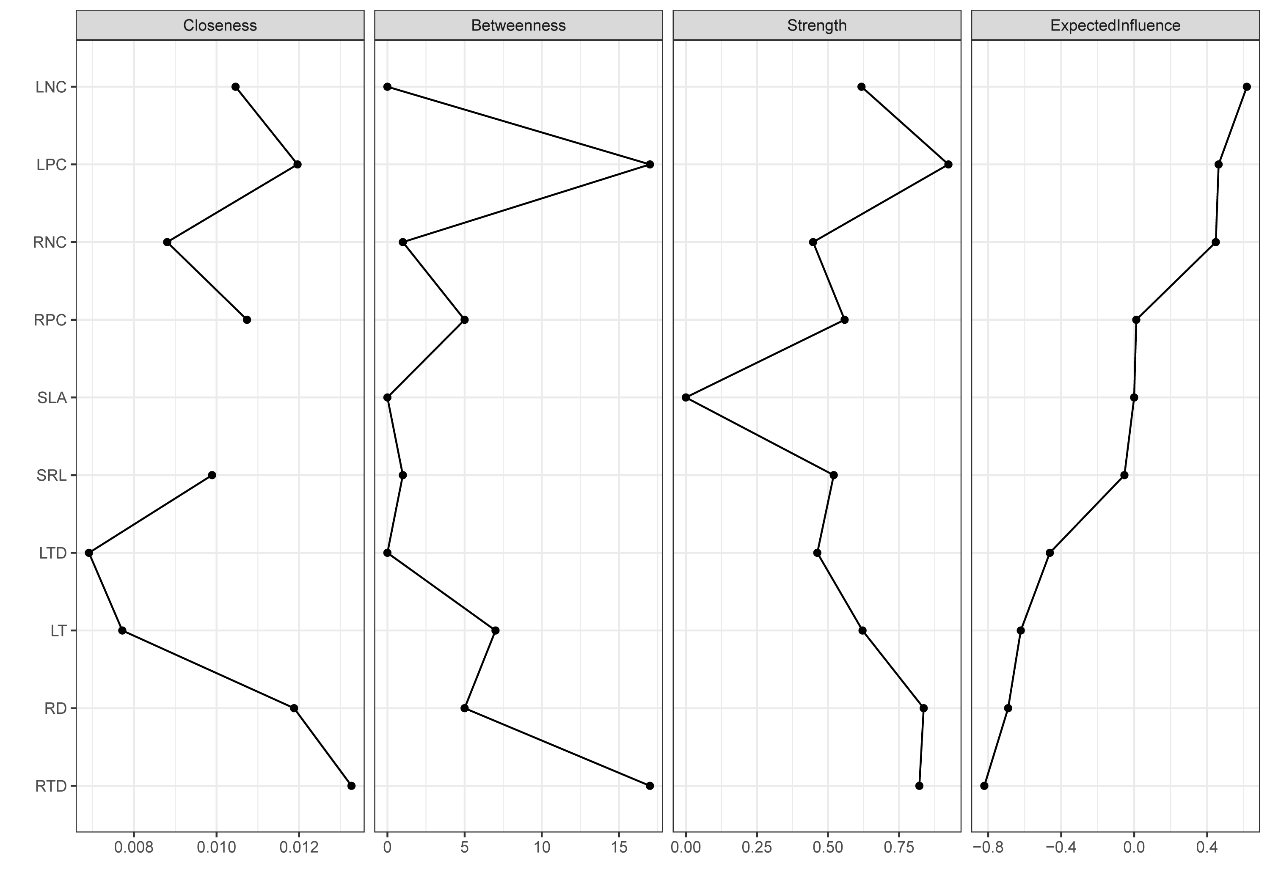


**Figure S4.** Centrality parameters of the bamboos in the network ordered by the indicator of Expected Influence. Closeness centrality indicates the distance between a node and all the other nodes in the network. Betweenness centrality indicates the normalized number of all shortest paths going through the node in the network. Strength centrality indicates the sum of all the edge weights of a node in the network. Expected Influence measures the influence of a node in the overall network by calculating the expected influence of each node on the other nodes. Nodes having higher expected influence in the network are considered to be more important. See Table 2 for trait abbreviations.


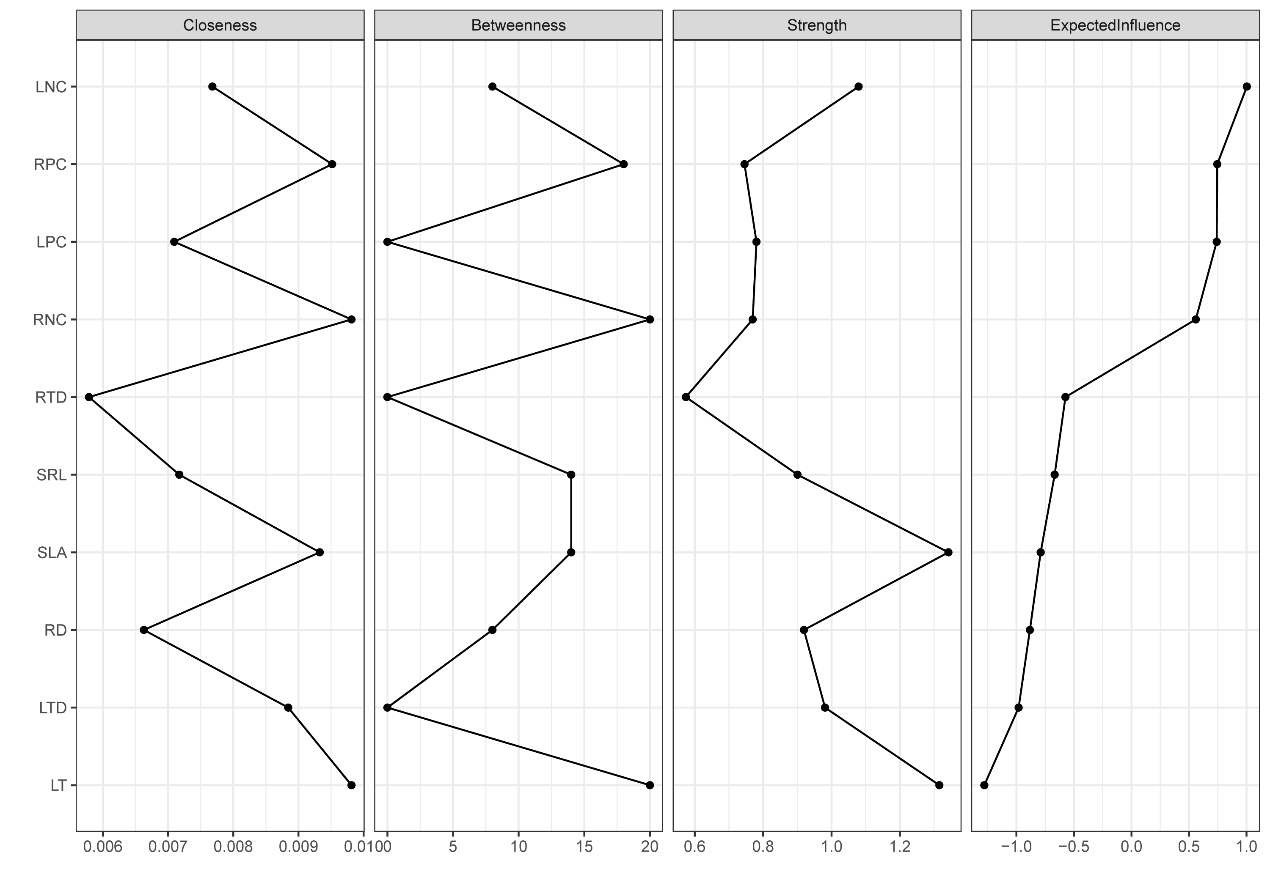


**Figure S5.** Centrality parameters of the broadleaf species in the network ordered by the indicator of Expected Influence. See Table 2 for trait abbreviations.
